# 

**Published:** 1895-12

**Authors:** 


### CONTENTS.

ORIGINAL ARTICLES—

Remarks on Goitre, with Report of Cases. By Dr. Joseph A. White, A. M., M. D., Richmond, Va. .......... 603

SOCIETY REPORTS—

Transactions of the Tri-State Medical Association—*Concluded*. . 610

SELECTIONS AND ABSTRACTS—

The Gold Combinations as Alternatives. By Thomas Hunt Stucky M. D., Ph. D., Louisville, Ky. .......... 622

Vulga-Vaginal Anus .......... 634

Anal-gesia and Sedation an Essential Adjunct to Treatment. By John J. Sullivan, New York ........ 635
Editorial ........ 637
Notes ........ 638
Book Reviews, Etc ........ 640
Books Received ........ 641
Index to Volume XXV ........ 642
Authors and Contributors ........ 643
Southern Medical Record Premium List ........ 644
Special Notes ........ 646
Prescriptions ........ 652

